# Expression of hMLH1 is inactivated in the gastric adenomas with enhanced microsatellite instability

**DOI:** 10.1054/bjoc.2001.2051

**Published:** 2001-10

**Authors:** M J Baek, H Kang, S E Kim, J H Park, J S Lee, Y-K Paik, H Kim

**Affiliations:** Departments of Pathology, Microbiology, Pediatrics, Yonsei Proteome Research Center, Brain Korea 21 Projects for Medical Sciences, Yonsei University College of Medicine, Seoul, Korea

**Keywords:** gastric adenoma, gastric carcinoma, microsatellite instability, hMLH1, hMSH2

## Abstract

Microsatellite instability (MSI) and frameshift mutations in the genes containing coding nucleotide repeats have been reported in a subset of gastric adenomas, however the inactivation profiles of DNA mismatch repair genes in MSI-positive gastric adenomas have not been characterized. To address the origin of MSI in gastric adenomas, expressions of hMLH1 and hMSH2 were explored in 86 gastric adenomas. Gastric carcinomas, of which 16 were MSI-positive and 22 MSI-negative, were used as controls. MSI was found in 15 (17%) of gastric adenomas. Absent or decreased hMLH1 expression by immunohistochemistry was noted in most of the MSI-positive adenomas (13/15, 87%) and carcinomas (14/16, 88%), and all of these tumours showed methylation of the *hMLH1* gene promoter. In contrast, rare inactivation of hMLH1 expression was found in MSI-negative adenomas (3/71, 4%) and carcinomas (2/22, 9%). Intense expression of hMSH2 gene product was observed in most of the gastric adenomas and carcinomas regardless of MSI status. These findings indicate that the inactivation of hMLH1 gene expression by promoter methylation is an early event and might be the origin of MSI-positive gastric adenomas. © 2001 Cancer Research Campaign  http://www.bjcancer.com


*British Journal of Cancer* (2001) **85**(8), 1147–1152
© 2001 Cancer Research Campaign
doi: 10.1054/ bjoc.2001.2051, available online at http://www.idealibrary.com on IDEAL

http://www.bjcancer.com

### Expression of hMLH1 is inactivated in the gastric adenomas with enhanced microsatellite instability

**MJ Baek[1,5], H Kang[1], SE Kim[1], JH Park[2,5], JS Lee[3], Y-K Paik[4] and H Kim[1,4,5]**

Departments of [1]Pathology, [2]Microbiology, [3]Pediatrics, [4]Yonsei Proteome Research Center, [5]Brain Korea 21 Projects for Medical Sciences, Yonsei University College of Medicine, Seoul, Korea

**Summary** Microsatellite instability (MSI) and frameshift mutations in the genes containing coding nucleotide repeats have been reported in a subset of gastric adenomas, however the inactivation profiles of DNA mismatch repair genes in MSI-positive gastric adenomas have not been characterized. To address the origin of MSI in gastric adenomas, expressions of hMLH1 and hMSH2 were explored in 86 gastric adenomas. Gastric carcinomas, of which 16 were MSI-positive and 22 MSI-negative, were used as controls. MSI was found in 15 (17%) of gastric adenomas. Absent or decreased hMLH1 expression by immunohistochemistry was noted in most of the MSI-positive adenomas (13/15, 87%) and carcinomas (14/16, 88%), and all of these tumours showed methylation of the *hMLH1* gene promoter. In contrast, rare inactivation of hMLH1 expression was found in MSI-negative adenomas (3/71, 4%) and carcinomas (2/22, 9%). Intense expression of hMSH2 gene product was observed in most of the gastric adenomas and carcinomas regardless of MSI status. These findings indicate that the inactivation of hMLH1 gene expression by promoter methylation is an early event and might be the origin of MSI-positive gastric adenomas. © 2001 Cancer Research Campaign http://www.bjcancer.com

**Keywords**: gastric adenoma; gastric carcinoma; microsatellite instability; hMLH1; hMSH2

Alterations in the size of microsatellite DNA sequences, namely microsatellite instability (MSI), have been demonstrated in a subset of gastrointestinal carcinomas (Aaltonen et al, 1993; Han et al, 1993; Ionov et al, 1993; Thibodeau et al, 1993). MSI results from the inactivation of DNA mismatch repair (MMR) genes. The defective MMR system does not correct the heteroduplex DNA formed due to slippage-induced replication errors, and insertions and deletions of repeat units occur after subsequent replication (Strand et al, 1993). Among the MMR genes, the inactivation of hMSH2 and hMLH1 genes are most frequently observed in colorectal carcinomas with MSI (Kinzler and Vogelstein, 1996; Thibodeau et al, 1996; Peltomaki et al, 1997). Recent studies demonstrated frequent hMLH1 gene inactivation in the tumours with MSI and most of the inactivation was related to the methylation of the *hMLH1* promoter (Kane et al, 1997; Cunningham et al, 1998).

Gastric adenoma is a precancerous lesion and a subset of gastric adenomas show MSI (Tamura et al, 1995; Semba et al, 1996; Kim et al, 1999). We and others have previously reported that the incidence of MSI in gastric adenomas was similar to that of gastric carcinomas, however the frameshift mutations at the coding mononucleotide repeats were confined to several genes and the mutational rate was much lower in the MSI positive gastric adenomas (Kim et al, 1999). Frequent inactivation of the hMLH1 gene by promoter methylation has been reported in MSI-positive gastric carcinomas (Fleischer et al, 1999; Leung et al, 1999), but the origin of MSI in gastric adenomas has not been characterized. In this study, we performed an immunohistochemical study for the two mismatch repair genes, hMSH2 and hMLH1, and examination of the *hMLH1* gene promoter methylation on gastric adenomas with or without MSI and compared the results with that of gastric carcinomas. Our results demonstrate that MSI in gastric adenomas originates from inactivation of hMLH1 by promoter methylation.

*Received 24 April 2001*
*Revised 21 June 2001*
*Accepted 11 July 2001*

*Correspondence to:* H Kim

### MATERIALS AND METHODS

### Patients and tissue samples

In total, 86 cases histologically confirmed as gastric adenoma and 38 gastric carcinomas were included in this study. Among them, 56 gastric adenomas and 18 gastric carcinomas had been analysed previously for MSI status and frameshift mutation of the target genes (Kim et al, 1999). All the adenoma cases were identified in the Department of Pathology at Yonsei University Medical Center between September 1995 and February 2000. All cases were examined by 3 pathologists (HKa, SEK and HKi) without knowledge of the molecular data. Among the 86 gastric adenomas, 16 cases had separate synchronous carcinoma and 7 cases had multiple synchronous adenomas. The 16 MSI-positive gastric carcinomas and 22 MSI-negative carcinomas were selected for comparison from the ongoing study of 113 cases. DNAs were extracted from tissue sections from surgical specimens that had been fixed in formalin and embedded in paraffin for routine histopathological examination. To enrich the tumour cell population, sections with more than 70% of tumour cell areas were selected from the haematoxylin-eosin-stained slides and isolated by microdissection.

### Screening of microsatellite instability

DNAs from gastric tumours and paired normal DNAs were PCR amplified at 5 microsatellite loci (*BAT26*, *BAT25*, *D2S123*, *D5S346*, and *D17S250*) to evaluate the MSI (Boland et al, 1998). PCR reactions were carried out as described previously (Kim et al, 1999). MSI was determined by the mobility shift of products from PCR. Based on the number of markers displaying instability per tumour, cases showing MSI in two or more markers were classified as MSI-positive and the rest of the cases were classified as MSI-negative (Figure 1A).

### Frameshift mutation analysis of the target genes

Frameshift mutations of the 5 target genes (the region comprising the (A) 10 tract in the *TGF-β RII* gene, (G)8 tract in the *BAX* gene, (A)8 tract of the *hMSH3* gene, (C)8 tract of the *hMSH6* gene and (G)8 tract of the *IGFIIR* gene) in our 15 cases of MSI-positive gastric adenomas and 16 cases of MSI-positive gastric carcinomas were analysed by PCR amplification as described previously (Kim et al, 1999).

### Immunohistochemical analysis of hMLH1 and hMSH2

Formalin-fixed and paraffin-embedded tissues were used for the immunostaining of hMLH1 and hMSH2. Deparaffinization and rehydration were performed using xylene and alcohol. The sections were treated with 0.3% hydrogen peroxidase for 3 min and blocking antibody for 30 min. Mouse monoclonal antibodies against hMLH1 protein (clone G168-728, PharMingen, San Diego, CA) and hMSH2 protein (clone G219-1129, PharMingen) were used in combination with antigen retrieval in a citrate buffer. Avidin–biotin complex methodology was employed. The chromogen was diaminobenzidine and counterstaining was done with haematoxylin. The evaluation of hMSH2 and hMLH1 gene product expression was categorized as positive, decreased or negative (Figure 2). Cases with definite nuclear staining in more than 30% of the tumour cells were categorized as positive, cases with definite nuclear staining in less than 30% of the tumour cells were categorized as decreased and cases with complete absence of nuclear staining were categorized as negative.

### Methylation analysis of *hMLH1* gene

We investigated the status of *hMLH1*-promoter region in 35 gastric adenomas (15 MSI-positive gastric adenomas and 20 MSI-negative gastric adenomas) and 38 gastric carcinomas (16 MSI-positive gastric carcinomas and 22 MSI-negative gastric carcinomas). DNA methylation patterns in the promoter region of *hMLH1* gene were determined by chemical modification of unmethylated DNA and subsequent PCR using primers specific for either methylated or modified unmethylated DNA. Two sets of primers were used for our methylation analysis (Table 1). One set was designed to amplify the region of –256 to –92 relative to the transcriptional start site, and the forward primer contained 3 CpG sites within the specific region in the *hMLH1* promoter reported to specifically control hMLH1 expression (Deng et al, 1999). The other set of primers was designed to amplify the region from –76 to +47 relative to the transcriptional start site, as previously described (Yanagisawa et al, 2000). The promoter methylation was regarded as positive when any of the 2 regions of the promoter was methylated by PCR-based promoter methylation assay. The chemical modification of unmethylated DNA with sodium bisulfite treatment was performed essentially as described previously with minor modification (Herman et al, 1996).

### RESULTS

### MSI status in gastric adenomas

We found MSI-positive adenomas (MSI was demonstrated at more than 2 of the 5 examined markers) in 15 (17%) of 86 gastric adenomas. Representative examples of gastric adenomas and carcinomas with MSI are shown in Figure 1A.

The MSI status in the synchronous adenoma and carcinoma was mostly the same, however, different MSI status was also found. Among the 16 synchronous adenomas and carcinomas, 5 cases showed MSI in both, and one case showed MSI only in the adenoma (Table 2).

### Frameshift mutations of the target genes

We analysed frameshift mutations by PCR amplification of the region comprising the polynucleotide tract in the *TGF-β RII* gene, *BAX* gene, *hMSH3* gene, *hMSH6* gene and *IGFIIR* gene in the gastric adenomas and carcinomas with MSI. The frameshift mutations of these 5 examined target genes were much less frequent in the gastric adenomas than the gastric carcinomas ($P = 0.019$ by $\chi^2$ test, Table 3). In the 15 MSI-positive gastric adenomas, 5 cases showed no frameshift mutations from the 5 target genes. Frameshift mutations in one gene were noted in 8 cases, 2 genes in 1 case and 3 genes in 1 case. Among the 16 MSI-positive gastric carcinomas, no frameshift mutation was noted in only one

**Table 1** PCR primers used for hMLH1 promoter methylation analysis

| Genomic position[a] | Primer set[b] | Primer sequences | Size (bp) | Annealing temp (°C) |
|---|---|---|---|---|
| –256 ~ –92 | U sense | AGGAAGAGTGGATAGTGATTTTTAAT | 165 | 56 |
| | antisense | CAACCCCACCCTTCAACA | | |
| –251 ~ –92 | M sense | GAGCGGATAGCGATTTTTAAC | 160 | 58 |
| | antisense | CAACCCCACCCTTCAACG | | |
| –76 ~ +47 | U sense | AGTTGAAGGAAGAATGTGAGTAT | 124 | 61 |
| | antisense | CAAATAACCCCTACCACAAACA | | |
| –75 ~ +46 | M sense | GTTGAAGGAAGAACGTGAGTAC | 122 | 63 |
| | antisense | GAATAACCCCTACCACGAACG | | |

[a]Genomic position is the location of the 5′ nucleotide of the sense primer in relation to the major transcriptional start site defined in the Genbank accession number, U40960. [b]U, unmethylation-specific primer; M, methylation-specific primer.

 *© 2001 Cancer Research Campaign*

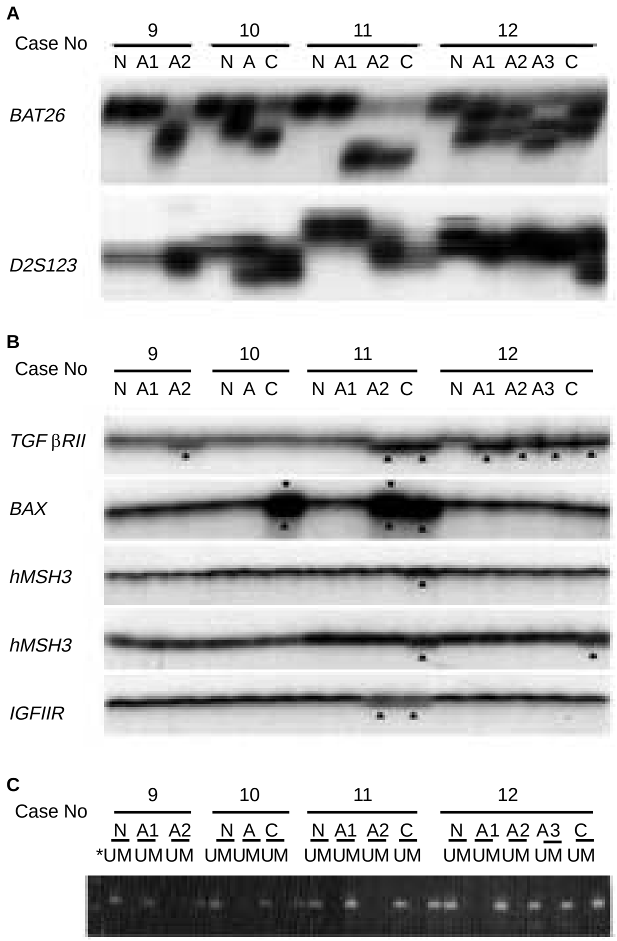

**Figure 1** Representative examples of microsatellite instability (**A**), frameshift mutations of the 5 target genes (**B**), and promoter methylation of *hMLH1* gene (**C**) in the gastric adenomas and carcinomas. Case 9 had two gastric adenomas, and cases 10, 11 and 12 had synchronous adenoma(s) and carcinoma. In each case the adenoma (A), carcinoma (C) and corresponding normal mucosa tissue (N) are shown. (**A**) MSI at *BAT26* and *D2S123* was found in the adenoma #2 of case 9, adenoma and carcinoma of case 10, adenoma #2 and carcinoma of case 11, and all the adenomas and carcinoma of case 12. (**B**) Frameshift mutations at coding nucleotide repeats of the 5 target genes in the gastric adenomas and carcinomas. Abnormal bands with base insertions and deletions and indicated (arrowhead). Frameshift mutations are found only in the cases of MSI-positive gastric adenomas and carcinomas and additional mutations of *hMSH3* and *hMSH6* were found only in the carcinoma of case 11 and 12. (**C**) Methylation analysis of *hMLH1* promoter. All the cases with MSI showed product from the PCR reaction with the methylation-specific primers. *, 100 bp ladder size marker; U, PCR products from unmethylated DNA of *hMLH1* promoter region; M, PCR products from methylated DNA of *hMLH1* promoter region case. Frameshift mutation in one gene was noted in 4 cases, 2 genes in 7 cases, 3 genes in one case, 4 genes in 2 cases and all 5 genes in one case (Table 3). Increased frameshift mutations of these 5 target genes in the carcinomas were also found in the cases of synchronous adenomas and carcinomas (Table 2). All the mutations in the adenoma were present in the coexistent carcinoma and several additional mutations were present only in the carcinoma (Figure 1B).

### Methylation status of *hMLH1*

The methylation status of *hMLH1*-promoter region was analysed in 15 MSI-positive gastric adenomas, 20 MSI-negative gastric adenomas, 16 MSI-positive gastric carcinomas and 22 MSI-negative gastric carcinomas. The expression of hMLH1 was directly related to the methylation status of the *hMLH1* promoter (Table 3 and Figure 1C). Among these 73 gastric tumours, 30 cases showed no or decreased hMLH1 expression (13 MSI-positive adenomas, 1 MSI-negative adenoma, 14 MSI-positive carcinomas and 2 MSI-negative carcinomas) and the remaining 43 cases showed intense hMLH1 expression. In the 30 gastric tumours with no or decreased hMLH1 expression, all showed *hMLH1* promoter methylation; 26 cases showed methylation in both examined regions and 4 cases showed methylation in only one region. Among the 43 gastric tumours with strong hMLH1 expression, no methylation was found in 34 cases, methylation in one region was found in 7 cases and methylation in both regions were found in 2 cases.

### Expression of hMLH1 and hMSH2 in gastric adenomas

Immunohistochemically, the hMLH1 protein was expressed in the nucleus of normal and tumour tissues. In normal tissue, most of the epithelial cells in the crypt, some stromal cells in the lamina propria and many lymphocytes in the lymphoid follicle showed nuclear staining. In tumour tissue, the intensity of expression was different according to the MSI status. In 15 MSI-positive adenomas, no hMLH1 expression in the tumour was observed in 7 cases and decreased expression was observed in 6 cases and the remaining 2 cases showed intense nuclear staining (Figure 2). In the 71 MSI-negative adenomas, 68 cases showed strong hMLH1 expression and 3 cases showed decreased expression in the nucleus of the tumour cells. The expression pattern of hMLH1 in MSI-positive gastric carcinomas was identical to that of gastric adenomas. Among the 16 MSI-positive gastric carcinomas, 11 showed no hMLH1 expression, 3 cases showed decreased expression and the remaining 2 cases showed intense nuclear expression (Figure 2). In contrast, 20 out of 22 MSI-negative carcinomas showed intense nuclear expression of hMLH1 and the remaining 2 cases showed decreased expression.

The expression of hMSH2 was also observed in normal and tumour tissues. In normal tissue, the hMSH2 protein expression was exclusively nuclear and was observed in the normal epithelium of the mucous neck and in the germinal centre of the lymphoid follicle. In tumour tissues, most of the cases showed intense nuclear staining. No different expression pattern according to the MSI status was observed. Intense nuclear staining was demonstrated in 14 (93%) out of 15 MSI-positive tumours and in 68 (95%) out of 71 MSI-negative adenomas. Therefore, the hMSH2 protein was expressed in most of the gastric carcinomas irrespective of MSI status (Table 3).

### DISCUSSION

In this study, we have analysed the expression of 2 members of the MMR gene, *hMLH1* and *hMSH2*, in gastric adenomas. We report the striking difference in the expression of the *hMLH1* gene according to the MSI status of the gastric adenomas. We also report that the abnormal hMLH1 expression in gastric adenoma is related to the methylation status of the *hMLH1* gene promoter.

There have been many studies demonstrating MSI in gastric adenomas and carcinomas (Rhyu et al, 1994; Lin et al, 1995; Tamura et al, 1995; Chung et al, 1996; Semba et al, 1996; Wu et al, 1998; Kim et al, 1999). The MSI pathway begins with inactivation of one of a group of genes responsible for DNA nucleotide

© 2001 Cancer Research Campaign

**Table 2** Immunohistochemical and mutational profiles of synchronous MSI-positive gastric adenomas and carcinomas

| Type of tumour | MSI[a] status | Expression of[b] | | Promoter methylation of *hMLH1* | Frameshift mutations[c] | | | | |
|---|---|---|---|---|---|---|---|---|---|
| | | hMSH2 | hMLH1 | | *TGFβ RII* | *BAX* | *hMSH3* | *hMSH6* | *IGFIIR* |
| Case 10 | | | | | | | | | |
| Adenoma | + | + | – | + | | | | | |
| Carcinoma | + | + | – | + | | +1/–1 | | | |
| Case 11 | | | | | | | | | |
| Adenoma #1 | – | + | + | – | | | | | |
| Adenoma #2 | + | + | – | + | –1/–1 | +1/–1 | | | –1/w |
| Carcinoma | + | + | – | + | –1/–1 | –1/w | –1/w | –1/w | –1/w |
| Case 12 | | | | | | | | | |
| Adenoma #1 | + | + | – | + | –1/–1 | | | | |
| Adenoma #2 | + | + | – | + | –1/w | | | | |
| Adenoma #3 | + | + | – | + | –1/–1 | | | | |
| Carcinoma | + | + | – | + | –1/–1 | | | –1/w | |
| Case 13 | | | | | | | | | |
| Adenoma | + | + | – | + | –2/–2 | | | | |
| Carcinoma | + | + | – | + | –1/–2 | | | | +1/w |
| Case 14 | | | | | | | | | |
| Adenoma | + | + | – | + | | | –1/w | | |
| Carcinoma | + | + | – | + | –1/–2 | | –1/w | | |
| Case 15 | | | | | | | | | |
| Adenoma | + | + | – | + | | | | –1/w | |
| Carcinoma | – | + | + | – | | | | | |

[a]microsatellite instability; +, presence of MSI; –, no instability. [b]+, expression; –, absent or decreased expression. [c]Number of inserted (+) or deleted (–) base pairs on each allele; w, wild type.

**Table 3** Immunohistochemical and genetic characteristics of MSI-positive gastric adenomas and carcinomas

| Variables | Categories | No. of MSI-positive[b] adenomas (*n* = 15) | No. of MSI-positive carcinomas (*n* = 16) | *P* value |
|---|---|---|---|---|
| hMSH2 expression | absent | 1 | 0 | NS[c] |
| | present | 14 | 16 | |
| hMLH1 expression | absent | 13 | 14 | NS |
| | present | 2 | 2 | |
| *hMLH1* promoter methylation | absent | 1 | 1 | NS |
| | present | 14 | 15 | |
| Number of target genes with frameshift mutations[a] | | | | 0.019 |
| | 0 | 5 | 1 | |
| | 1 | 8 | 4 | |
| | 2 | 1 | 7 | |
| | 3 | 1 | 1 | |
| | 4 | 0 | 2 | |
| | 5 | 0 | 1 | |

[a]frameshift mutations of the polynucleotide repeats of the *TGFβ RII, BAX, hMSH3, hMSH6* and *IGFIIR* genes was analysed. [b]microsatellite instability-positive. [c]not significant ($P > 0.05$).

mismatch repair, leading to extensive mutations in the repetitive DNA sequences with low frequencies of allelic losses and rare alteration of tumour DNA content (Loeb, 1994). Recent studies have delineated that up to 90% of sporadic MSI-positive colorectal carcinomas are due to the inactivation of *hMLH1* gene, principally transcriptional silencing, and the remainder are consistent with inactivation of hMSH2 and hMLH1 by somatic mutation (Kane et al, 1997; Herman et al, 1998; Thibodeau et al, 1998). Studies on endometrial carcinomas also have shown a significant correlation between MSI and hypermethylation of the *hMLH1* gene promoter (Esteller et al, 1998, 1999; Gurin et al, 1999; Simpkins et al, 1999). Highly frequent inactivation of hMLH1 gene has also been reported in MSI-positive gastric carcinomas. Leung et al (1999) found 100% hypermethylation of *hMLH1* gene promoter in 11 MSI-positive gastric carcinomas and that 10 cases (90%) showed complete absence of hMLH1 protein in the tumour cells. Other studies demonstrated promoter methylation of *hMLH1* gene in 3 of 5 (60%, Toyota et al, 1999), 14 of 18 (78%, Fleisher et al, 1999) and 5 of 8 (63%, Suzuki et al, 1999) MSI-positive gastric carcinomas. In this study, we demonstrated loss of or decreased hMLH1 expression in 13 of 15 (87%) MSI-positive gastric adenomas and 14 of 16 (88%) MSI-positive gastric carcinomas. We also found that all of those cases showed promoter hypermethylation of the *hMLH1* gene. These results suggest that transcriptional silencing of the *hMLH1* gene is the causative genetic event both in the MSI-positive gastric adenomas and carcinomas and that

 *© 2001 Cancer Research Campaign*

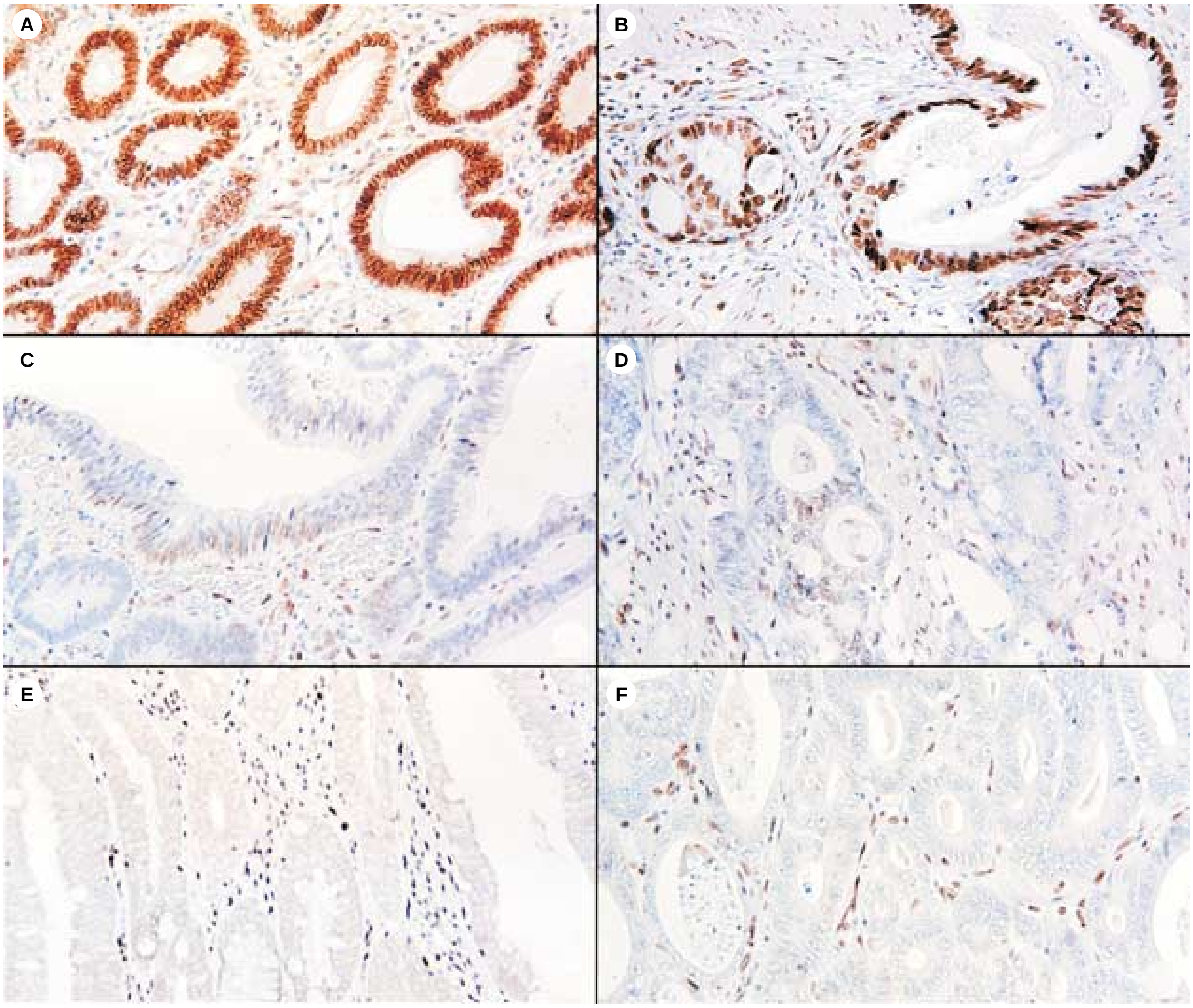

**Figure 2** Expression of hMLH1 in the gastric adenomas and carcinomas. Positive hMLH1 expression is noted in the nucleus of the gastric adenoma (**A**) and carcinoma (**B**) cells. Decreased hMLH1 expression in the nucleus of gastric adenoma (**C**) and carcinoma (**D**) cells and complete loss of hMLH1 expression in gastric adenoma (**E**) and carcinoma (**F**) cells are noted, whereas adjacent stromal cells and scattered lymphoid cells are positive for hMLH1 expression this inactivation is an early genetic event in the gastric carcinomas with MSI-positive phenotype.

In our MSI-positive gastric adenomas and carcinomas, most of the tumour cells showed complete loss of hMLH1 expression, though some cases showed decreased hMLH1 expression compared to the normal epithelial cells. This decreased hMLH1 expression was also found in some of the MSI-negative adenomas and carcinomas. This incomplete loss of hMLH1 expression might result from monoallelic hypermethylation or hypermethylation in some proportion of the tumour cells, however differentiating between monoallelic and biallelic inactivation is not always possible in studies using human samples. Another possibility for this incomplete hMLH1 inactivation lies in the methylation status of the promoter. A recent study suggests that the area of promoter methylation of the *hMLH1* promoter region is critical for hMLH1 inactivation (Deng et al, 1999). In this experiment, we evaluated 2 different regions in the promoter, and one of which contained 3 CpG islands in the specific sites reported to control hMLH1 expression. In our gastric adenomas and carcinomas, some cases showed different methylation status for these 2 regions. We could not, however, find the specific relationship between the inactivation of hMLH1 expression and region-specific promoter hypermethylation, because many cases with methylation in one of the 2 regions also showed complete loss of hMLH1 expression. Inversely, some rare cases of MSI-positive tumours (one adenoma and one carcinoma) with *hMLH1* promoter methylation in both regions showed positive hMLH1 expression. From this experiment, however, we could not explain the mechanisms of these rare exceptional cases. To address this issue more precisely, the overall consequence of changes after hypermethylation of *hMLH1* promoter on gene expression should be elucidated in the future.

We demonstrated the same inactivation pattern of the MMR genes in the synchronous MSI-positive gastric adenomas and carcinomas. Among the 16 synchronous gastric adenomas and carcinomas, 5 cases showed MSI both in the adenomas and carcinomas. In these synchronous MSI-positive gastric adenomas and carcinomas, both adenomas and carcinomas showed loss of hMLH1 expression and promoter hypermethylation of *hMLH1* gene. There were no differences in the number of markers with MSI between the adenomas and carcinomas, however frameshift

*© 2001 Cancer Research Campaign* 

mutations of the target genes were more frequent in the coexistent carcinomas. These findings are in accordance with our previous results that more frameshift mutations of the genes containing mononucleotide repeats were present in the carcinomas. The results presented here support the hypothesis that the inactivation of hMLH1 and MSI is an early event before or during the formation of the gastric adenomas and accumulated frameshift mutations are late events related to the adenoma progression and malignant transformation.

### ACKNOWLEDGEMENTS

We are grateful for Haeryoung Kim and Juwon Kim for assistance in English. We also thank Dr Chung Mo Nam for statistical analysis. This work was supported by the 21C Frontier Functional Genomics (to YKP: Grant No. FG-1-4-01).

### REFERENCES

Aaltonen LA, Peltomaki P, Leach FS, Sistonen P, Pylkkanen L, Mecklin JP, Jarvinen H, Powell SM, Jen J, Hamilton SR, Petersen GH, Kinzler KW, Vogelstein B and de la Chapelle A (1993) Clues to the pathogenesis of familial colorectal cancer. *Science (Washington, DC)* **260**: 812–816

Boland CR, Thibodeau SN, Hamilton SR, Sidransky D, Eshleman JR, Burt RW, Meltzer SJ, Rodriguez-Bigas MA, Fodde R, Ranzani GN and Srivastava S (1998) A national cancer institute workshop on microsatellite instability for cancer detection and familial predisposition: Development of international criteria for the determination of microsatellite instability in colorectal cancer. *Cancer Res* **58**: 5248–5257

Chung YJ, Song JM, Lee JY, Jung YT, Seo EJ, Choi SW and Rhyu MG (1996) Microsatellite instability-associated mutations associate preferentially with the intestinal type of primary gastric carcinomas in a high-risk population. *Cancer Res* **56**: 4662–4665

Cunningham JM, Christensen ER, Tester DJ, Kim CY, Roche PC, Burgart LJ and Thibodeau SN (1998) Hypermethylation of the *hMLH1* promoter in colon cancer with microsatellite instability. *Cancer Res* **58**: 3455–3460

Deng G, Chen A, Hong J, Chae HS and Kim YS (1999) Methylation of CpG in a small region of the hMLH1 promoter invariably correlates with the absence of gene expression. *Cancer Res* **59**: 2029–2033

Esteller M, Levine R, Baylin SB, Ellenson LH and Herman JG (1998) *MLH1* promoter hypermethylation is associated with the microsatellite instability phenotype in sporadic endometrial carcinomas. *Oncogene* **16**: 2413–2417

Esteller M, Catasus L, Matias-Guiu X, Mutter GL, Prat J, Baylin SB and Herman JG (1999) *hMLH1* promoter hypermethylation is an early event in human endometrial tumorigenesis. *Am J Pathol* **155**: 1767–1772

Fleisher AS, Esteller M, Wang S, Tamura G, Suzuki H, Yin J, Zou TT, Abraham JM, Kong D, Smolinski KN, Shi YQ, Rhyu MG, Powell SM, James SP, Wilson KT, Herman JG and Meltzer SJ (1999) Hypermethylation of the *hMLH1* gene promoter in human gastric cancers with microsatellite instability. *Cancer Res* **59**: 1090–1095

Gurin CC, Federici MG, Kang L and Boyd J (1999) Causes and consequences of microsatellite instability in endometrial carcinoma. *Cancer Res* **59**: 462–466

Han HJ, Yanagisawa A, Kato Y, Park JG and Nakamura Y (1993) Genetic instability in pancreatic cancer and poorly differentiated type of gastric cancer. *Cancer Res* **53**: 5087–5089

Herman JG, Graff JR, Myohanen S, Nelkin BD and Baylin SB (1996) Methylation-specific PCR: a novel PCR assay for methylation status of CpG islands. *Proc Natl Acad Sci USA* **93**: 9821–9826

Herman JG, Umar A, Polyak K, Graff JR, Ahuja N, Issa JP, Markowitz S, Willson JK, Hamilton SR, Kinzler KW, Kane MF, Kolodner RD, Vogelstein B, Kunkel TA and Baylin SB (1998) Incidence and functional consequences of *hMLH1* promoter hypermethylation in colorectal carcinoma. *Proc Natl Acad Sci USA* **95**: 6870–6875

Ionov Y, Peinado MA, Malkhosyan S, Shibata D and Perucho M (1993) Ubiquitous somatic mutations in simple repeated sequences reveal a new mechanism for colonic carcinogenesis. *Nature* **363**: 558–561

Kane MF, Loda M, Gaida GM, Lipman J, Mishra R, Goldman H, Jessup JM and Kolodner R (1997) Methylation of the *hMLH1* promoter correlates with lack of expression of hMLH1 in sporadic colon tumors and mismatch repair-defective human tumor cell lines. *Cancer Res* **57**: 808–811

Kim JJ, Baek MJ, Kim L, Kim NG, Lee YC, Song SY, Noh SH and Kim H (1999) Accumulated frameshift mutations at coding nucleotide repeats during the progression of gastric carcinoma with microsatellite instability. *Lab Invest* **79**: 1113–1120

Kinzler KW and Vogelstein B (1996) Lessons from hereditary colorectal cancer. *Cell* **87**: 159–170

Leung SY, Yuen ST, Chung LP, Chu KM, Chan AS and Ho JC (1999) *hMLH1* promoter methylation and lack of *hMLH1* expression in sporadic gastric carcinomas with high-frequency microsatellite instability. *Cancer Res* **59**: 159–164

Lin JT, Wu MS, Shun CT, Lee WJ, Wang JT, Wang TH and Sheu JC (1995) Microsatellite instability in gastric carcinoma with special references to histopathology and cancer stages. *Eur J Cancer* **31A**: 1879–1882

Loeb LA (1994) Microsatellite instability: marker of a mutator phenotype in cancer. *Cancer Res* **54**: 5059–5063

Peltomaki P, Vasen HF and The International Collaborative Group on Hereditary Nonpolyposis Colorectal Cancer (1997) Mutations predisposing to hereditary nonpolyposis colorectal cancer: database and results of a collaborative study. *Gastroenterology* **113**: 1146–1158

Rhyu MG, Park WS and Meltzer SJ (1994) Microsatellite instability occurs frequently in human gastric carcinoma. *Oncogene* **9**: 29–32

Semba S, Yokozaki H, Yamamoto S, Yasui W and Tahara E (1996) Microsatellite instability in precancerous lesions and adenocarcinomas of the stomach. *Cancer* **77**: 1620–1627

Simpkins SB, Bocker T, Swisher EM, Mutch DG, Gersell DJ, Kovatich AJ, Palazzo JP, Fishel R and Goodfellow PJ (1999) MLH1 promoter methylation and gene silencing is the primary cause of microsatellite instability in sporadic endometrial cancers. *Hum Mol Genet* **8**: 661–666

Strand M, Prolla TA, Liskay RM and Petes TD (1993) Destabilization of tracts of simple repetitive DNA in yeast by mutations affecting DNA mismatch repair. *Nature* **365**: 274–276

Suzuki H, Itoh F, Toyota M, Kikuchi T, Kakiuchi H, Hinoda Y and Imai K (1999) Distinct methylation pattern and microsatellite instability in sporadic gastric cancer. *Int J Cancer* **83**: 309–313

Tamura G, Sakata K, Maesawa C, Suzuki Y, Terashima M, Satoh K, Sekiyama S, Suzuki A, Eda Y and Satodate R (1995) Microsatellite alterations in adenoma and differentiated adenocarcinoma of the stomach. *Cancer Res* **55**: 1933–1936

Thibodeau SN, Bren G and Schaid D (1993) Microsatellite instability in cancer of the proximal colon. *Science (Washington, DC)* **260**: 816–819

Thibodeau SN, French AJ, Roche PC, Cunningham JM, Tester DJ, Lindor NM, Moslein G, Baker SM, Liskay RM, Burgart LJ, Honchel R and Halling KC (1996) Altered expression of hMSH2 and hMLH1 in tumors with microsatellite instability and genetic alterations in mismatch repair genes. *Cancer Res* **56**: 4836–4840

Thibodeau SN, French AJ, Cunningham JM, Tester D, Burgart LJ, Roche PC, McDonnell SK, Schaid DJ, Vockley CW, Michels VV, Farr GH, Jr. and O'Connell MJ (1998) Microsatellite instability in colorectal cancer: Different mutator phenotypes and the principal involvement of *hMLH1*. *Cancer Res* **58**: 1713–1718

Toyota M, Ahuja N, Suzuki H, Itoh F, Ohe-Toyota M, Imai K, Baylin SB and Issa JP (1999) Aberrant methylation in gastric cancer associated with the CpG island methylator phenotype. *Cancer Res* **59**: 5438–5442

Wu MS, Lee CW, Shun CT, Wang HP, Lee WJ, Sheu JC and Lin JT (1998) Clinicopathological significance of altered loci of replication error and microsatellite instability-associated mutations in gastric cancer. *Cancer Res* **58**: 1494–1497

Yanagisawa Y, Akiyama Y, Iida S, Ito E, Nomizu T, Sugihara K, Yuasa Y and Maruyama K (2000) Methylation of the *hMLH1* promoter in familial gastric cancer with microsatellite instability. *Int J Cancer* **85**: 50–53

 *© 2001 Cancer Research Campaign*
